# Effect of High-Intensity Interval Training on Cardiac Apoptosis Markers in Methamphetamine-Dependent Rats

**DOI:** 10.3390/cimb44070209

**Published:** 2022-07-04

**Authors:** Hadi Shahrabadi, Amir Hossein Haghighi, Roya Askari, Majid Asadi-Shekaari, Daniel Costa Souza, Paulo Gentil

**Affiliations:** 1 Department of Exercise Physiology, Faculty of Sport Sciences, Hakim Sabzevari University, Sabzevar 9617976487, Iran; h.shahrabadi@gmail.com (H.S.); r.askari@hsu.ac.ir (R.A.); 2 Neuroscience Research Center, Neuropharmacology Institute, Kerman University of Medical Sciences, Kerman 7619813159, Iran; majidasadi@kmu.ac.ir; 3 College of Physical Education and Dance, Federal University of Goias, Goiania 74690-900, Brazil; daniel_souza86@hotmail.com (D.C.S.); paulogentil@hotmail.com (P.G.); 4 Hypertension League, Federal University of Goias, Goiania 74690-900, Brazil; 5 Instituto VIDA, Brasilia 70673-464, Brazil

**Keywords:** interval training, heart, cell death, drug abuse, aerobic exercise, exercise is medicine

## Abstract

Chronic methamphetamine use increases apoptosis, leading to heart failure and sudden cardiac death. Previous studies have shown the importance of high-intensity interval training (HIIT) in reducing indices of cardiac tissue apoptosis in different patients, but in the field of sports science, the molecular mechanisms of apoptosis in methamphetamine-dependent rats are still unclear. The present article aimed to investigate the changes in cardiac apoptosis markers in methamphetamine-dependent rats in response to HIIT. Left ventricular tissue was used to evaluate caspase-3, melusin, FAK, and IQGAP1 gene expression. Rats were divided into four groups: sham, methamphetamine (METH), METH-control, and METH-HIIT. METH was injected for 21 days and then the METH-HIIT group performed HIIT for 8 weeks at 5 sessions per week. The METH groups showed increased caspase-3 gene expression and decreased melusin, FAK, and IQGAP1 when compared to the sham group. METH-HIIT showed decreased caspase-3 and increased melusin and FAK gene expression compared with the METH and METH-control groups. The IQGAP1 gene was higher in METH-HIIT when compared with METH, while no difference was observed between METH-HIIT and METH-control. Twenty-one days of METH exposure increased apoptosis markers in rat cardiac tissue; however, HIIT might have a protective effect, as shown by the apoptosis markers.

## 1. Introduction

Methamphetamine (METH) is a sympathomimetic amine that can be consumed by smoking, inhalation, ingestion, or injection [1]. Chronic METH use causes cardiomyopathy [2] with increased cardiac cells apoptosis, which might lead to heart failure and death [3].

Some molecular markers related to apoptosis factors are proteases called caspases, which degrade the nuclear and cytoskeletal proteins, resulting in cell death [4,5]. Among the members of the caspase family, caspase-3 expression has been shown to induce transient depression in cardiac function and abnormal nuclear and myofibrillar ultrastructural damage, leading to increases in infarct size and a pronounced susceptibility to death in rats [6].

Melusin is a chaperone protein expressed specifically in cardiac and skeletal muscles [7]. Melusin helps to regulate natural heart rhythm and contraction, as well as reduce infiltration of inflammatory cells, fibrosis, and cardiomyocyte apoptosis, thus helping to preserve heart morphology and function [8,9]. In cardiomyocytes, melusin interacts with other signaling proteins that work in these pathways, including focal adhesion kinase (FAK), IQ-motif-containing GTPase activating protein 1 (IQGAP1), c-Raf, mitogen-activated/extracellular signal–regulated protein kinase kinases 1/2 (MEK1/2), extracellular-signal-regulated kinase 1/2 (ERK1/2), and phosphatidylinositol 3 kinase (PI3K) [10,11]. FAK and IQGAP1 are both integrin pathway proteins. FAK is involved in the activation of ERK1/2 in response to mechanical stretch, and IQGAP1 is a scaffold protein capable of binding to many other proteins, including c-Raf, MEK1/2, and ERK1/2, facilitating their sequential phosphorylation [10]. Previous studies have shown that FAK and IQGAP1 with melusin have protective effects against myocardial damage and apoptosis [12,13,14].

Previous studies have shown that 14 days of METH injection (1 to 5 mg/kg) reduced protein expression of melusin, FAK, and IQGAP1, and increased cleaved caspase-3 expression in rats [15]. Based on this, it can be suggested that melusin might be a mediator of cardiac tissue damage induced by METH. On the other hand, non-pharmacological methods, especially exercise training, has been shown to prevent and treat different cardiovascular diseases [16] and to prevent cardiomyocyte death in many different circumstances [17,18,19]. Among the different exercise modalities, high-intensity interval training (HIIT) has been gained increased attention for its potential cardiovascular benefits [20,21,22].

The mechanisms of exercise-induced cardio protection, especially against apoptosis, are not fully understood; however, molecular analysis might help to explore the potential mechanisms, such as apoptosis-related protein expression, decreased release of mitochondrial apoptogenic factors, and changes in reactive oxygen species (ROS) and antioxidant status [23]. In this regard, Lu et al. [24] analyzed the effects of HIIT on apoptosis, oxidative stress, and metabolism in rats with an infarcted myocardium, showing reduced caspase-3 gene expression. In contrast, Banaei et al. [25] showed that rats with myocardial ischemia–reperfusion injury showed no reduction in caspase-3 protein in response to HIIT. Moreover, Wolff et al. [26] observed that 10 weeks of training on a treadmill increased the melusin mRNA concentration in rats with myocardial infarction and pericardiectomy.

Considering the medical and social burden of METH use, it would be important to explore the molecular aspects of potential strategies to prevent damage to health [27], especially to the heart. Therefore, the aim of the present study was to investigate the effects of 8 weeks of HIIT on caspase-3, melusin, FAK, and IQGAP1 gene expression in cardiac tissue of METH-dependent rats.

## 2. Materials and Methods

### 2.1. Study Design and Animals

**Figure 1:**
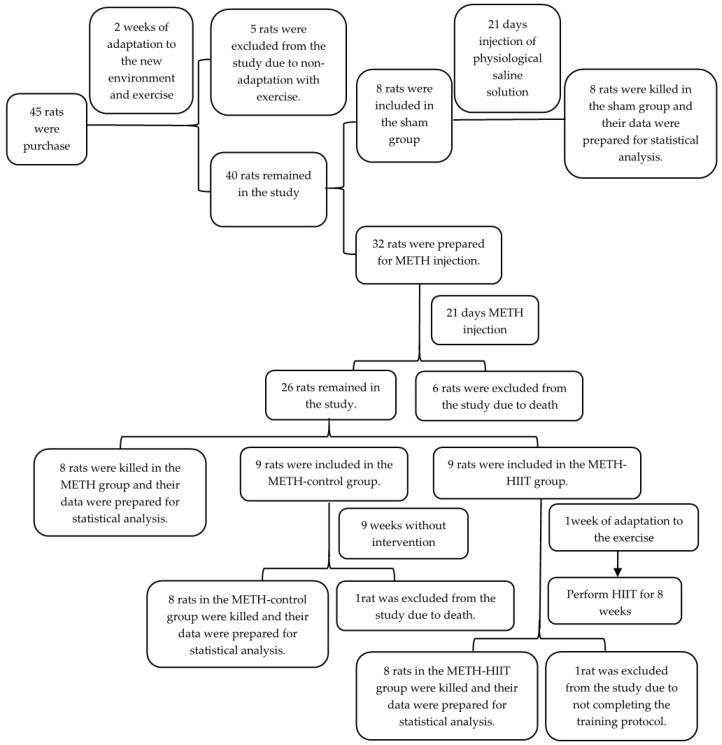
Study design. METH: methamphetamine; HIIT: high-intensity interval training.

The present study is experimental and fundamental research. The study was approved by the University Ethics Committee and followed ethical principles regarding how to work with laboratory animals. Forty-five male Wistar rats, weighing between 180 and 220 g, were purchased from an animal farm. We opted to use only male rats because the hormonal fluctuations that occur in females might interfere with our intervention and tests. Rats were kept at room temperature (21 ± 2 °C) and 40–60% humidity under a 12-h sleep–wake cycle and free access to standard food pellets and water. Rats were divided into four groups—sham, METH, METH-control, and METH-HIIT—using a simple random method (lottery), as shown in Figure 1.

### 2.2. METH Injection

During 21 consecutive days, rats from the METH, METH-control, and METH-HIIT groups were injected with 5 mg/kg METH dissolved in physiological saline solution (0.9% sodium chloride) [15,28]. The sham group received 0.5 mL of physiological saline solution After 21 days, rats from the METH and sham groups were sacrificed and heart tissue was extracted. METH-HIIT and METH-control continued the intervention for eight more weeks.

### 2.3. Exercise Training Protocol

The METH-HIIT group underwent a one-week familiarization period before starting the training intervention. During this period, rats exercised on a treadmill for 10 min at 10 to 20 m/min per session [25]. Electric shock (0.5 mA) was also used for stimulating rats to run. Two days after the last familiarization session, they performed a maximum incremental treadmill test. The test started at 10 m/min and increased 3 m/min every 3 min [29]. HIIT was performed at 85% of the maximum speed with no inclination. HIIT was performed five days per week for eight weeks on a rodent treadmill, as shown in Table 1.

Each training session started with six minutes of warm-up and ended with six minutes cool down at 8 m/min. Rats in the METH-control group were placed on a turned-off treadmill 5 times a week for 5 to 10 min per session to have the same environmental stress conditions as the METH-HIIT group.

### 2.4. Tissue Extraction

Tissue extraction was performed at two moments. First, after 21 days of METH or sham injection for the sham and METH groups. Second, 24 h after the last training session for the METH-control and METH-HIIT groups. Rats were anesthetized with CO_2_ gas and then sacrificed by decapitation. The hearts were immediately excised, and then the left ventricle was separated. Left ventricular tissue samples were transferred to microtubes, frozen in liquid nitrogen, and stored at −70 °C to evaluate the caspase 3, melusin, FAK, and IQGAP1 genes’ expression.

### 2.5. Primer Design and Synthesis

The primer sequences of the caspase-3, melusin, FAK, IQGAP1, and GAPDH (Glyceraldehyde-3-Phosphate Dehydrogenase) genes used in this study were designed by Primer-Blast (NCBI) online software (Table 2) and synthesized by Sinaclon Co., Tehran, Iran.

### 2.6. RNA Extraction and cDNA Synthesis

RNA extraction was performed using TRIzol solution (Yekta Tajhiz Azma, Tehran, Iran) according to the manufacturer′s protocol for all samples. For this purpose, 50 mg of left ventricular tissue was lysed in TRIzol solution. For extracting the RNA, chloroform and isopropanol was used, which was then washed with 75% ethanol. All samples were analyzed by a Picodrop device (Picodrop limited, Hinxton, UK) to evaluate the quantity and quality of the RNA extracted. The cDNA synthesis was performed by the reverse transcription method from RNA extracted using a cDNA synthesis kit (Cat No: YT4500, Yekta Tajhiz Azma, Tehran, Iran) and based on the cDNA synthesis protocol included in the kit.

### 2.7. Real Time RT-PCR

Genes expression levels was measured by Real-Time PCR (qRT-PCR) (Rotor Gene Q, Qiagen, Germany). Real Q Plus 2x Master Mix Green-high Rox™ (Ampliqon, Denmark), cDNA, and synthesized primers were used for this step. The temperature profile was as follows: initial denaturation at 95 °C for 15 min followed by 40 consecutive cycles of denaturation at 95 °C for 10 s, annealing at 60 °C for 20 s, and extension at 72 °C for 20 s. The amplification curve of each PCR reaction was normalized with the amplification curve of the GAPDH reference gene. The 2^−∆∆CT^ formula was also used to determine the gene expression in the present study.

### 2.8. Statistical Analysis

A Shapiro–Wilk test was used to check the normality of the data. Welch′s ANOVA test was used to examine the differences between groups and Dunnett′s T3 multiple comparisons test was used to determine the differences between pairs of groups. All the statistical analyses were performed using GraphPad Prism software, version 8, and a *p*-value < 0.05 was considered significant.

## 3. Results

The result of the Welch′s ANOVA showed a significant difference between the sham, METH, METH-control, and METH-HIIT groups for caspase-3 (*p* = 0.0014), melusin (*p* < 0.0001), FAK (*p* < 0.0001), and IQGAP1 (*p* < 0.0001) genes expression.

**Figure 2:**
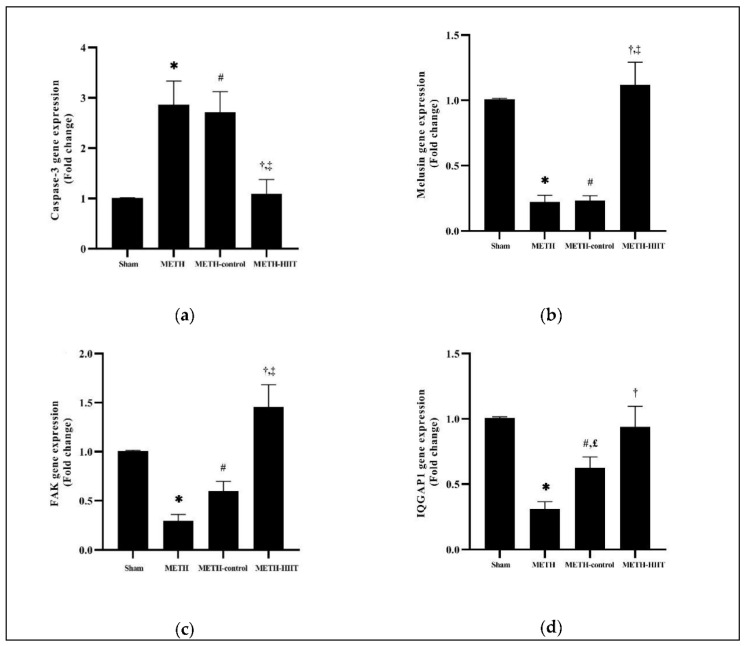
Effects of METH injection and HIIT program on the gene expression levels in the left ventricle (the data are presented as the mean ± SEM): (**a**) caspase-3 gene expression; (**b**) melusin gene expression; (**c**) FAK gene expression; (**d**) IQGAP1 gene expression. *, significant differences between the METH and sham groups; #, significant differences between the METH-control and sham groups; †, significant differences between the METH-HIIT and METH groups; ‡, significant differences between the METH-HIIT and METH-control groups; £, significant differences between the METH-control and METH groups. METH: methamphetamine; HIIT: high-intensity interval training; FAK: focal adhesion kinase; IQGAP1: IQ-motif-containing GTPase activating protein 1.

Dunnett′s T3 multiple comparisons showed that caspase-3 gene expression significantly increased for the METH group when compared to the sham group (*p* = 0.0266). Caspase-3 expression in METH-HIIT was significantly lower than in METH (*p* = 0.0381) and METH-control (*p* = 0.0319). The METH-control group’s caspase-3 expression was higher than that of the sham group (*p* = 0.0193) (Figure 2a).

Melusin gene expression was significantly decreased for METH when compared to the sham group (*p* < 0.0001). Cardiac melusin gene expression was higher in METH-HIIT compared to METH (*p* = 0.0062) and METH-control (*p* = 0.0058). Melusin gene expression for METH-control was lower than for the sham group (*p* < 0.0001) (Figure 2b).

Dunnett′s T3 multiple comparisons showed that METH had significantly lower FAK gene expression when compared with the sham group (*p* < 0.0001). FAK gene expression was significantly increased in the METH-HIIT group compared with METH (*p* = 0.0063) and METH-control (*p* = 0.0324). The expression of this gene in the METH-control group was lower than for the sham group (*p* = 0.0226) (Figure 2c).

Dunnett′s T3 multiple comparisons test showed that after 21 days of METH injection, IQGAP1 gene expression in the METH group was significantly reduced when compared to the sham group (*p* < 0.0001). After 8 weeks of HIIT, IQGAP1 expression in METH-HIIT was significantly higher than in METH (*p* = 0.0254), but similar to METH-control (*p* = 0.4569). IQGAP1 gene expression was higher in the METH-control group than in METH (*p* = 0.0409) and lower than the sham group (*p* = 0.0121) (Figure 2d).

## 4. Discussion

The present study aimed to investigate changes in cardiac apoptosis markers in METH-dependent rats after eight weeks of high-intensity interval training. The results showed that 21 days of METH injection increased the caspase-3 gene expression and decreased the expression of melusin, FAK, and IQGAP1 genes. These results are in agreement with Chen et al. [30] who showed that METH can stimulate cardiomyocyte apoptosis in vitro and in vivo. Moreover, Liou et al. [3] suggested that chronic METH use increased cardiac apoptosis. Sun et al. [15] showed that melusin and the proteins related to apoptosis, such as FAK and IQGAP1, and its downstream effectors—phosphorylated AKT, ERK, and GSK3β—decreased in isolated cardiomyocytes and cardiomyocytes of rats exposed to METH. Our results bring additional support to the suggestion that melusin and its related proteins play an important role in the apoptosis signaling pathway, such that increasing melusin gene expression preserves anti-apoptotic pathways in cardiac tissue.

The mechanisms and pathological responses of the cardiovascular system to METH use remain largely unknown. However, previous research suggest that METH can stimulate catecholamines secretion, mitochondrial dysfunction and ROS production [31,32]. The decrease in melusin in response to METH might be associated with catecholamines [15], impaired gene expression and protein synthesis. Moreover, ROS produced by mitochondrial damage can also influence melusin.

Our results show that eight weeks of HIIT decreased the gene expression of caspase-3 but increased melusin, FAK, and IQGAP1 gene expression. However, IQGAP1 gene expression was not significantly increased in METH-HIIT when compared to the METH group.

To the best of our knowledge, no previous study has examined the apoptosis signaling pathway in response to METH and HIIT. However, the results of the present study agree with those of Delfan et al. [33], Lu et al. [24], and Wolff et al. [26]. Delfan et al. [33] and Lu et al. [24] suggested that HIIT reduces caspase-3 gene expression in rat models of diabetes and myocardial infarction. Additionally, Wolff et al. [26] showed that treadmill training (15° inclination and 22 m/min) increases the melusin mRNA concentration in myocardial infarction and pericardiectomy mice.

Decreased apoptosis might be related to increased phosphorylated AKT and ERK1/2, increased phosphorylated GSK3β, and its inactivation by increasing these two factors. These might lead to suppression of pro-apoptotic protein expression, such as BCL-2-associated death promoter (BAD) and BCL-2-associated X protein (BAX), and increase the anti-apoptotic protein expression, such as B cell leukemia-2 (BCL-2) [34]. Moreover, BCL-2 family proteins control the integrity of the mitochondrial outer membrane and prevent the release of apoptosis-stimulating proteins [32].

The increased melusin level in the left ventricle might be related to the mechanical stress created on the heart during high-intensity exercise [26]. Melusin is a membrane receptor that connects the intracellular cytoskeleton with the extracellular matrix and allows muscle cells to respond to mechanical stimulation [7]. A significant increase in IQGAP1 gene expression was observed in the METH-control group compared to the METH group, which is probably due to the improvement in heart tissue in rats due to lack of methamphetamine use.

HIIT might also reduce cardiac apoptosis by reducing oxidative stress, increasing antioxidant indices [24,35], and promoting a better autonomic balance [36]. Lu et al. [24] showed that HIIT reduces the concentration of malondialdehyde (MDA) and increases the antioxidant markers, such as superoxide dismutase (SOD) and glutathione peroxidase (GPx), in rats with myocardial infarction. These results are associated with decreased genes expression of caspase-3 and BAX and increased BCl-2 gene expression. Silva et al. [36] showed that 8 weeks of HIIT promoted a reduction in cardiac sympathetic modulation, which might lead to a reduction in catecholamine release. Another factor that may reduce the rate of cardiac apoptosis due to HIIT is insulin-like growth factor-1 (IGF-1), as shown by Delfan et al. [33].

The present study has limitations that researchers should consider in future studies: not evaluating other indicators of the apoptosis signaling pathway, including AKT, ERK1/2, and GSK3β; not evaluating proteins associated with apoptotic markers by the Western blot method; do not have immunohistochemical data to show apoptosis. Moreover, it would be interesting to perform future studies with larger sample sizes to confirm the results.

## 5. Conclusions

Molecular analysis suggested that HIIT seems to be a non-pharmacological method to reduce cardiomyocyte apoptosis induced by METH. The present study might help to pave the way for further and comprehensive research in this field. Based on the present results, it is expected that addiction treatments and rehabilitation centers could use HIIT to counteract cardiomyocyte apoptosis in METH users and similar clinical conditions.

## Figures and Tables

**Table 1:** Characteristics of the high-intensity interval training.

Weeks	1	2	3	4	5	6	7	8
Number of intervals	4	4	4	4	4	4	4	4
Effort duration (min)	2	2	2	2	2	2	2	2
Effort velocity (m/min)	22	24	26	28	30	32	34	36
Rest duration	2	2	3	3	4	4	4	4
Rest velocity (m/min)	10	10	11	11	12	12	13	13

**Table 2:** Primer sequences in this research.

Gene Name	Forward Primer	Reverse Primer
Caspase-3	5′-GCAGCAGCCTCAAATTGTTGACTA-3′	5′-TGCTCCGGCTCAAACCATC-3′
Melusin	5′-GGGTGAAGGCCAGTCAAACT-3′	5′-TGCTCCACGTTTATGACCCC-3′
FAK	5′-CTTAATCTGGCCAGGACGGT-3′	5′-GAAGCACGGTTTGAGAGGTG-3′
IQGAP1	5′-ACAATCTGGAGACGCAAGCA-3′	5′-AGCTGCTCTCGGTTATACGC-3′
GAPDH	5′-CAACTCCCTCAAGATTGTCAGCAA-3′	5′-GGCATGGACTGTGGTCATGA-3′

FAK: focal adhesion kinase; IQGAP1: IQ-motif-containing GTPase activating protein 1; GAPDH: glyceraldehyde-3-phosphate dehydrogenase.

## Data Availability

The data is available under reasonable request to the corresponding author.
